# Epithelial-to-Mesenchymal Plasticity Harnesses Endocytic Circuitries

**DOI:** 10.3389/fonc.2015.00045

**Published:** 2015-02-26

**Authors:** Salvatore Corallino, Maria Grazia Malabarba, Martina Zobel, Pier Paolo Di Fiore, Giorgio Scita

**Affiliations:** ^1^Fondazione Istituto FIRC di Oncologia Molecolare (IFOM), Milan, Italy; ^2^Dipartimento di Scienze della Salute, Università degli Studi di Milano, Milan, Italy; ^3^Dipartimento di Oncologia Sperimentale, Istituto Europeo di Oncologia, Milan, Italy

**Keywords:** endocytic pathway, epithelial junctions remodeling, endocytosis and EMT, EMT and cancer, WNT and TGF-β signaling

## Abstract

The ability of cells to alter their phenotypic and morphological characteristics, known as cellular plasticity, is critical in normal embryonic development and adult tissue repair and contributes to the pathogenesis of diseases, such as organ fibrosis and cancer. The epithelial-to-mesenchymal transition (EMT) is a type of cellular plasticity. This transition involves genetic and epigenetic changes as well as alterations in protein expression and post-translational modifications. These changes result in reduced cell-cell adhesion, enhanced cell adhesion to the extracellular matrix, and altered organization of the cytoskeleton and of cell polarity. Among these modifications, loss of cell polarity represents the nearly invariable, distinguishing feature of EMT that frequently precedes the other traits or might even occur in their absence. EMT transforms cell morphology and physiology, and hence cell identity, from one typical of cells that form a tight barrier, like epithelial and endothelial cells, to one characterized by a highly motile mesenchymal phenotype. Time-resolved proteomic and phosphoproteomic analyses of cells undergoing EMT recently identified thousands of changes in proteins involved in many cellular processes, including cell proliferation and motility, DNA repair, and – unexpectedly – membrane trafficking (1). These results have highlighted a picture of great complexity. First, the EMT transition is not an all-or-none response but rather a gradual process that develops over time. Second, EMT events are highly dynamic and frequently reversible, involving both cell-autonomous and non-autonomous mechanisms. The net results is that EMT generates populations of mixed cells, with partial or full phenotypes, possibly accounting (at least in part) for the physiological as well as pathological cellular heterogeneity of some tissues. Endocytic circuitries have emerged as complex connectivity infrastructures for numerous cellular networks required for the execution of different biological processes, with a primary role in the control of polarized functions. Thus, they may be relevant for controlling EMT or certain aspects of it. Here, by discussing a few paradigmatic cases, we will outline how endocytosis may be harnessed by the EMT process to promote dynamic changes in cellular identity, and to increase cellular flexibility and adaptation to micro-environmental cues, ultimately impacting on physiological and pathological processes, first and foremost cancer progression.

## Introduction

The epithelial-to-mesenchymal transition (EMT) is a fundamental process in embryonic development and tissue repair. EMT is key also for the progression of diseases, including organ fibrosis and cancer (2–4). The pioneering work in the 1980 of Elizabeth Hay first described an “epithelial-mesenchymal transformation” using a model of chick primitive streak formation (5). Subsequently, the term “transformation” was replaced with “transition,” reflecting in part the reversibility of the process and the fact that it is distinct from neoplastic transformation (6, 7). The phenotypic plasticity associated with EMT is revealed by the occurrence of the reverse process, the mesenchymal-epithelial transition (MET), which involves the conversion of mesenchymal cells to epithelial derivatives.

Epithelial cells form polarized sheets that are held together by various cell adhesion molecules. Beneath this cell layer, the basement membrane anchors epithelial cells to the underlying matrix and maintains apical-basal polarity. Adhesion to both the basement membrane and adjacent cells is critical for maintaining the epithelial phenotype (8).

During EMT, cells lose these epithelial characteristics, acquiring instead an invasive and migratory mesenchymal phenotype, which allows them to leave the tissue parenchyma, undergo morphogenetic programs, generate new tissues during development or repair wounded ones, and to enter the blood circulation during cancer metastasis (2, 4).

A number of distinct molecular and cellular processes are engaged in order to initiate an EMT and to enable it to reach completion. These include activation of transcription factors, such as SNAIL, TWIST, ZEB, and others which are generally termed EMT-TFs (9), expression of specific cell-surface proteins, reorganization and expression of cytoskeletal proteins, production of enzymes that can degrade the extracellular matrix, changes in the expression of specific microRNAs, and – unexpectedly – alterations in membrane trafficking. EMT factors can be used as biomarkers to demonstrate the passage of a cell through an EMT. Consistently, hallmarks of EMT include the loss of expression or function of E-cadherin and reduced abundance of tight junction proteins and cytokeratins, as well as concomitant increase in abundance of mesenchymal markers, such as vimentin, fibronectin, fibroblast-specific protein 1, α-smooth muscle actin, and N-cadherin (10).

Epithelial-to-mesenchymal transitions are encountered in three distinct biological settings that carry very different functional consequences. Type-I EMT is typically associated with implantation, embryo formation, and organ development where the goal is to generate diverse cell types that share common mesenchymal phenotypes (11, 12). Importantly, this type of EMT does not cause fibrosis or induce an invasive phenotype. The EMT associated with wound healing, tissue regeneration, and organ fibrosis is classified as type-II EMT and it represents a program associated with repair responses that generate fibroblasts and allow for tissue reconstruction following trauma and inflammatory injury. Type-II EMT is associated with acute and chronic inflammation and frequently results in organ fibrosis. Finally, type-III EMT occurs in neoplastic cells that have previously undergone genetic and epigenetic changes, specifically in genes (oncogenes and tumor suppressor genes) that favor clonal outgrowth and the development of localized tumors. These latter changes cooperate with the EMT regulatory circuitry to produce outcomes far different from those observed in the other two types of EMT. While the actual impact of type-III EMT in disease pathogenesis is still the object of an on-going debate (13, 14), recent studies implicate EMT in the generation of cancer stem cells within primary tumors that may be prone to metastasizing (15). Additionally, the role of EMT in stemness has become a topic of particular interest, since the production of induced pluripotent stem cells requires an initial MET (16, 17).

Epithelial-to-mesenchymal transition, and the reverse process of MET, should not be thought of as simple binary states. Emerging evidence argues that EMT is better described as an analogical spectrum of partial EMT states. These states are dynamically interconvertible, providing a degree of cellular plasticity that critical contributes to cellular adaptation to intrinsic and extrinsic micro-environmental cues. For example, in the embryo, multiple rounds of EMT and MET are necessary to complete gastrulation and primitive streak formation, highlighting the reversibility of this process (3). An initial de-differentiation to a mesenchymal phenotype enables cells to migrate and then to undergo MET to give rise to multiple different cell types in the notochord, somites, primordia of the urogenital system, and the splanchnopleura and somatopleura (3). Similar oscillations between EMT and MET may account for the ability of tumor cell to metastasize, whereby cells acquire mesenchymal migratory features to detach from the primary mass, to revert to an epithelial identity, similar to the original tumors from which they arose, once they reach the final metastatic niche (18).

The dynamic and reversible nature of the EMT programs, capable of responding to both cell-autonomous and non-autonomous stimuli, may be caused, in addition to changes in the transcriptional make-up of cells, also by the rewiring or the harnessing of different cellular circuitries, including endocytic networks. Indeed, endocytosis has emerged in recent years as a highly interconnected infrastructure of various cellular circuitries that is essential for the execution of different cellular programs (19), including those promoting a canonical EMT program and relying on the activation of WNT or TGF-β signaling. In general, signaling outputs are rendered interpretable to the cell by the resolution of the signal, in space and time, executed through endocytosis and membrane trafficking (19–22). Accordingly, activated membrane receptors are internalized and transported as cargoes onto endocytic vesicles. Receptor-loaded endosomes may act either as specialized and spatially confined signaling hubs or sorting station for the subsequent recycling of cargos back to the plasma membrane (PM) to initiate a new round of signaling or for directing cargos to lysosomal degradation to extinguish signals. This process ensures control over signal duration, intensity, and ultimately has a major impact on biological outputs. This concept is summarized in the term “endocytic matrix,” which we coined a few years ago to indicate the pervasiveness of endocytic control over virtually every aspect of the life of a cell (19–21). The importance of endocytic wirings in cell regulation is mirrored by the relevance of its subversion in pathological processes, including cancer (23–26).

In this framework, it is not surprising that EMT might exploit the diffuse interconnectivity of the “matrix” to execute part of its program, with particular regard to the ability of a cell to perceive, transduce, and adapt to soluble cues as well as spatial information, ultimately affecting cell polarity, cell-cell and cell-matrix interaction, motility, and invasiveness.

In this review, we will focus on a few examples, from an increasing body of emerging literature, to illustrate the notion that EMT programs in addition to relying on specific transcriptional factors to change cellular identity, frequently harness endocytic networks either to properly execute EMT signaling or to promote changes leading to the emergence of mesenchymal properties.

## Inducers of EMT Use Endocytic Networks for the Execution of Their Signaling Programs

It is well established that biochemical and biomechanical environmental cues can trigger the onset of EMT. Among the soluble cues, members of the TGF-β family of cytokines and WNT ligands are potent inducer of this trans-differentiation program in various physiological conditions, such as during embryonic development, and in pathological contexts, including inflammation-induced fibrosis, wound healing, and cancer progression (2, 4, 27). The biochemistry of TGF-β and WNT pathways is the subject of a large body of literature and it is well characterized (28–30). In particular, and of relevance to the subject of this review, it is emerging that the fine tuning of the signaling output is dependent on spatial constraints (the subcellular compartments of localization of the various members of the signaling cascade) and on the trafficking routes that the receptors for TGF-β and WNT ligands utilize following engagement by their ligands (31–36). We will utilize, therefore, the exemplar cases of TGF-β and WNT signaling to provide a conceptual framework of how endocytic networks might be used to modulate trans-differentiation programs.

### TGF-β signaling, EMT, and endocytosis

TGF-β, and other members of the TGF-β superfamily of cytokines such as BMP, signal to the cell by binding to cell-surface cognate receptor(s) endowed with serine/threonine kinase activity. This interaction triggers a signaling cascade relying on three classes of proteins, globally referred to as SMAD(s) (30, 36–38). The first class (R-SMADs for receptor-regulated SMADs) is directly activated by phosphorylation and comprises SMAD2, 3, which are directly activated by type-I TGF-β receptor, and SMAD1, 5, 8, which are substrates of activated BMP receptors. After phosphorylation, the R-SMADs associate with a common mediator (Co-SMAD), the SMAD4 protein, forming oligomeric complexes that translocate to the nucleus acting as transcription factors. The third class of SMADs, including SMAD6 and 7, is constituted by regulatory inhibitory (I-SMADs) proteins that recruit the SMURF ubiquitin ligase, thereby controlling SMAD ubiquitination, and therefore stability (39, 40).

The outcome of this relative simple signaling cascade is the onset of a canonical transcriptional program that promotes EMT. TGF-β is also known to trigger a non-canonical, SMAD-independent signaling pathway that impinges on the activation of ERK, AKT, and RHO-GTPases, which are thought to contribute critically to the acquisition of certain mesenchymal morphological features [for reviews of canonical and non-canonical TGF-β signaling see Ref. (30, 36–38)].

**Figure 1:**
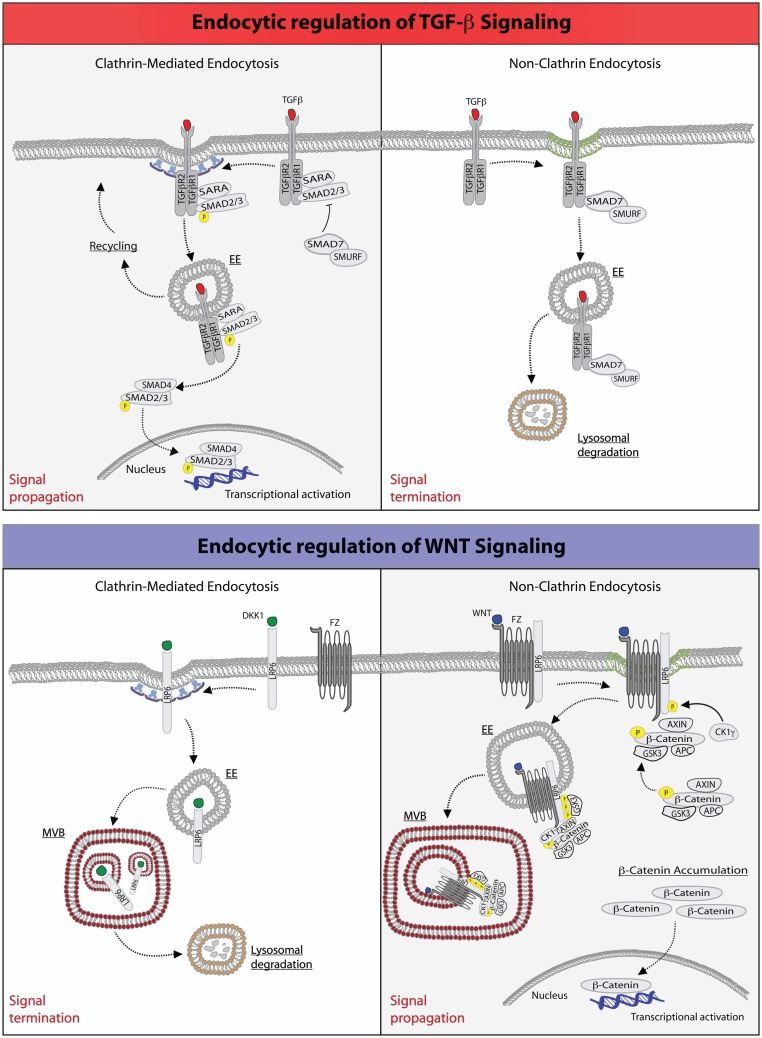
**Endocytic regulation of TGF-β and WNT pathways**. Top: endocytic regulation of TGF-β signaling. Left panel, upon ligand stimulation, type-I and type-II TGF-β-receptors form a heterodimeric complex (30, 36–38), which binds the SMAD2/3 and SARA proteins. Type-I TGF-β-receptor directly phosphorylates SMAD2/3, an event that may be promoted by the anchoring protein SARA (41–43, 50). SARA, in addition to facilitate SMAD2/3 phosphorylation at the PM, may also retain these proteins at this location retarding their release into the cytoplasm. Ligand-bound TGF-β-receptor is also rapidly internalized via CME into early endosome (EE) (41, 50). From this endocytic station, the receptor can be recycled back to the plasma membrane for a new round of signaling. Early endosomes, where SARA accumulates, may also serve as a mean to separate SARA from activated SMAD2/3, which would then be free to be released into the cytoplasm, where they form an oligomeric complex with SMAD4. The SMAD2/3-SMAD4 complex translocates, then, into the nucleus where it acts as a transcription factor ensuring execution of TGF-β signaling. Right panel, activation of TGF-β receptor(s) may also occur in cholesterol-rich membranes micro-domains (green membrane) (41, 50). This promotes the binding to the receptors of the SMAD7-SMURF2 complex followed by internalization into caveolae (51, 52). SMURF2 is an ubiquitin ligase that can ubiquitinate the receptor promoting its targeting into multivesicular bodies and its subsequent lysosomal degradation resulting in signal termination (51, 52). Bottom: endocytic regulation of WNT signaling. Left panel, upon binding of the antagonistic, WNT-like ligand, Dickkopf (DKK1), the low-density lipoprotein receptor-related 6 (LPR6) receptor is internalized by CME, and primarily transported through multivesicular body (MVB) to lysosomes for degradation, hence terminating signaling (33, 35). Right panel, the binding of WNT to the Frizzled (Fz) receptor, a seven-pass transmembrane receptor, promotes the formation of complex between Fz and LPR6 on cholesterol-rich lipid domain (green membrane). At this site, the cytoplasmic tail of LPR6 is phosphorylated by casein kinase 1γ (CK1γ) and glycogen synthase kinase 3 (GSK3), thereby activating NCE of the receptors (53). The components of the destruction complex – Axin, β-catenin, APC, and GSK3 – become bound to the activated and internalized Fz-LRP6 complex in endosomes. The subsequent transport of this assembly into MVBs may lead to the sequestration of the destruction complex into the internal vesicles of the MVB. Newly synthesized β-catenin may, thus, escape the destruction complex and accumulate into the cytoplasm to translocate into the nucleus and activate gene transcription (54).

Binding of TGF-β to its receptor, however, triggers also the internalization of the latter and its accumulation into specialized endosomal stations (Figure 1). More importantly, both the route of internalization and the ability to signal from endosomes affect the amplitude and duration of TGF-β signaling, ultimately impacting on the specificity of its biological outcome (35). For example, it is well established that TGF-β receptors are endocytosed through multiple internalization routes. The majority of the receptor is rapidly internalized via clathrin-mediated endocytosis (CME). TGF-β receptors, however, may also enter cells via non-clathrin endocytosis (NCE) routes, which rely on cholesterol-rich membrane micro-domains (lipid rafts/caveolae) (41–43). The partitioning on the PM, and hence the internalization routes, of TGF-β receptors can be regulated: interleukin-6 (IL6), ADAM metallopeptidase domain 12 (ADAM12), and the integrin-linked kinase (ILK) were reported to shift the receptors to the non-raft fractions (44–46), while cholesterol, heparan sulfate, and hyaluronan-CD44 promote the lipid raft/caveolae localization (47–49).

In other systems, such as the epidermal growth factor receptor (EGFR), the two endocytic routes have been shown to be linked to different fates of the internalized cargo, with CME leading to recycling of receptors to the PM, and NCE being associated to their routing to the lysosome for degradation (21). A similar situation is operational for TGF-β signaling, in which internalization plays both a stimulatory and an inhibitory role. In particular, it has been shown that CME into the EEA1-positive endosomes, where the SMAD2 anchor, SMAD anchor for receptor activation (SARA) is enriched, promotes TGF-β signaling (41, 50). In contrast, the lipid raft-caveolar internalization pathway contains the SMAD7-SMURF2-bound receptor and is required for rapid receptor turnover and termination of signaling (51, 52) (Figure 1).

The situation is however not as clear-cut as it may appear. In particular, it remains uncertain whether and how CME regulates SMAD2/3 phosphorylation in the receptor SARA-SMAD complex, which is presumably assembled at the PM. SARA contains both a SMAD-binding domain, which has been shown to interact with SMAD2 and SMAD3 (55), and a C-terminal region, which interacts with the receptor (50). Hence, SARA was proposed to play a key role in presenting R-SMADs to the receptor for phosphorylation and the ensuing signal propagation. By using the GM-CSFR-TGF-β-receptor fusion system in combination with endogenous TGF-β receptors, it was found that SARA is indeed crucial to bridge TGF-β receptors with SMAD2/3, and that the phosphorylation of receptors can take place even when CME is blocked; conversely, the receptor-mediated phosphorylation, and thus activation of SMAD2/3, requires receptor internalization (56). In a different system – constituted by human kidney mesangial cells – however, inhibition of CME only slightly affected TGF-β-induced SMAD2 phosphorylation and SMAD2-SMAD4 association, but decreased the nuclear accumulation of SMAD2, and therefore attenuated SMAD2-mediated transcriptional responses (57, 58). One possibility to reconcile these disparate findings is that SARA, at least in some cellular systems, may promote, but it is not essential for, the phosphorylation at the PM of SMAD2/3, which, however, would not be free to diffuse into the cytoplasm. Endocytosis may then serve as a mean to physically separate the activated, phosphorylated SMADs complex from SARA, which accumulates via internalization into endosomes, ultimately facilitating the nuclear translocation and transcriptional activity of SMADs. Within this context, inhibition of CME may aberrantly prolong the formation of SARA-SMAD2 complex at the PM, delaying its translocation to the nucleus.

A corollary of this scenario is that endosomal signaling should be critical to modulate the biochemical and biological outcome of TGF-β signaling. To this regard, it should be noted that SARA also contains a FYVE (Fab1, YOTB/ZK632.12, Vac1, and EEA1) domain. FYVE domains bind to phosphatidylinositol-3-phosphate (PI3P), a lipid enriched in early endosomes, and mediate the recruitment of FYVE-domain-containing proteins to early endosomes (59), which are enriched in PI3P. The FYVE domain has, consistently, been shown to be essential for the stimulatory effects of SARA on TGF-β signaling (50, 60), strengthening the notion that the localization in early endosomes is important to fulfill the functions of SARA. Endosomes, in this framework, may become important signaling centers, not only to regulate the life-time of SARA-SMAD2/3 complexes but also for the assembly of specific TGF-β-dependent multi-protein transducer complexes, as supported by findings that several positive regulators of TGF-β signaling are localized in endosomes. For instance, in addition to SARA, there are two additional FYVE domain-containing (and endosomally localized) proteins, endofin and hepatocyte growth factor-regulated tyrosine kinase substrate (HRS/HGS), which have all been suggested to promote signaling by TGF-β, as well as by other members of the TGF-β superfamily, such as activin and decapentaplegic (DPP) (50, 61–64).

Collectively this evidence points to an important regulatory role of endocytic factors in mediating TGF-β signaling, with potential diverse impact on the ability of the latter to induce EMT depending on the cellular context. Within this framework, it is not surprising that SARA, which as indicated above seems to have a prominent role in promoting TGF-beta signaling, may act in some context as suppressor of mesenchymal properties and markers (58). For example, while silencing of SARA has been shown to impair TGF-β signaling (50, 60), its aberrant expression may perturb endosomal trafficking, and impact on the formation and stability of proficient signaling complexes. These latter findings betray the fact that, while we have come to a detailed understanding of individual TGF-β signaling axis, it is still difficult to predict how perturbations of endocytic network impact on transcriptional and non-transcriptional-dependent TGF-β programs.

### WNT signaling, EMT, and endocytosis

One additional signaling axis that potently induces an overt EMT program is the WNT pathway (29). WNT signaling is initiated by soluble ligands of the WNT family that bind to the seven-pass transmembrane family of Frizzled (Fz) receptors and promote the subsequent formation of a trimeric complex with low-density-lipoprotein (LDL) receptor-related protein (LRP)6/5, single-span transmembrane proteins that belong to a subfamily of LRPs. This latter complex then recruits Axin, along with APC and GSK3β, the core components of the β-catenin destruction complex (65–67). The ensuing inactivation of the destruction complex results in the accumulation of β-catenin and the activation of its transcriptional target genes, which include a set of transcription factors such as *Snail*, *Slug*, and *Twist*, that regulate many of the cellular changes required for EMT (68–71).

Several mechanisms have been proposed for the inhibition of the destruction complex. All models agree that WNT signaling prevents GSK3β from phosphorylating existing or newly synthesized β-catenin, consistent with the finding that genetic removal or inhibition of GSK3β triggers signaling activity (16, 72, 73). However, the models differ in the mode of GSK3β inhibition, which could occur by disruption (of the integrity of the Axin/GSK3β/β-catenin), saturation (elevated expression of β-catenin may saturate the available, soluble destruction complex so that newly synthesized β-catenin molecules would be free to travel to the nucleus to exert their transcriptional activity), or sequestration, in endosomal lysosomal compartments, of the destruction complex (74, 75). In this latter case, WNT is thought to trigger endocytosis and trafficking of Fz and LRP6, along with associated GSK3β to the lumen of multivesicular bodies (MVBs; Figure 1). As a result, GSK3β would no longer have access to β-catenin and become unable to trigger its degradation (74), resulting in signal augmentation. Although the sequestration hypothesis has not been extensively tested, it provides a simple explanation as to how endocytosis could contribute to signaling since endocytosis is a prerequisite to MVB targeting.

Evidence that endocytosis contributes to canonical WNT signaling comes from experiments showing that pharmacological (76, 77) or genetic (78) blockade of internalization, obtained by impairing either the activity dynamin or by ablating CME, prevents WNT-mediated increase in β-catenin levels (79–81). Although this result argues that CME is required for WNT signaling, other investigations have suggested that, instead, caveolae-mediated endocytosis is key, perhaps by allowing LRP5/6 to accumulate in a lipid environment that promotes its phosphorylation (82, 83) (see also below and Figure 1). The latter suggestion is based on studies of Frizzled-5 (Fz-5), WNT3A, and LRP6 in cell culture (82). It was shown that when Fz-5 alone is expressed, it is internalized through CME, upon engagement by WNT3A. However, when both Fz-5 and LRP6 receptors are expressed, WNT addition causes Fz-5 colocalization with caveolin (Figure 1). Therefore, LRP6 may divert Fz/WNT complexes from a signaling-deficient clathrin-based route to a signaling-proficient caveolae-based pathway. The finding that Axin is recruited to the same vesicles corroborates the idea that the caveolae-associated complex is functionally significant for signaling. Furthermore, treatment with nystatin (a cholesterol-sequestering drug) or silencing of caveolin-1 prevents the WNT-dependent accumulation of β-catenin, while inhibition of CME has marginal effects (82). Thus, at least in some circumstances, CME may act to rapidly shut off WNT signaling, while caveolar endocytosis and the generation of cholesterol-rich PM-based platforms, may potentiate it: the opposite of what is seen with signaling by TGF-β (41) and by some ligands of receptor tyrosine kinases (84–87).

In addition to receptor levels also, the identity of the ligand is important in determining the route of internalization and the signaling outcome. It has been shown that WNT3A stimulates receptor uptake into caveolae, where LRP6 is phosphorylated by the kinase CK1γ. This modification primes LRP6 to promote the stabilization of β-catenin, as described above. Conversely, another WNT-like ligand, Dkk1, antagonizes this signaling route by triggering the internalization of the co-receptor via CME, a compartment that lacks CK1γ and thus suppresses β-catenin signals (33, 88).

While all this evidence points to a role for endocytosis in WNT signaling, it leaves the problem unresolved as to how (and how much) the different internalization routes contribute to it. While the discrepant results, relative to the importance of CME or caveolar endocytosis, might be ascribed to different cellular contexts, additional findings cloud the interpretation of the results. First, knockout mice for caveolin-1, 2, or 3 show no indication of WNT signaling deficiency (89). In addition, activated LRP6 has also been shown to form intracellular aggregates in response to WNT stimulation (83). The aggregates do not co-localize with fluid phase markers of endocytosis but do occasionally co-localize with caveolin. This implies that the formation of these aggregates may not strictly require endocytosis, albeit it is conceivable that they could form preferentially at the surface of caveolae, where specialized caveolin-based PM signaling platforms form. Within this framework, caveolin would improve the efficiency of signaling, but would not be absolutely necessary.

## Morphological and Functional Alterations Underlying EMT Harness Endocytic Routes: The Exemplar Case of E-Cadherin

Reduced cell-cell adhesion, enhanced cell adhesion to the extracellular matrix, and altered organization of the cytoskeleton and of cell polarity, with ensuing changes in cell shape, are nearly invariable features of early EMT. These changes frequently precede the full onset of genetic reprograming, albeit they may also represent the biological end point of the transcriptional modulation of genes directly involved in the above-mentioned processes. A case in point is represented by the adherence junction (AJ) proteins, E-cadherin and N-cadherin. Indeed, E-cadherin loss and N-cadherin gain are frequent events underlying the dissolution of cell-cell contacts during EMT (90). E-cadherin (encoded by the gene CDH1) loss, in most cases, is caused epigenetically by hyper-methylation of its promoter or, genetically, by active repression exerted by EMT transcription factors, which conversely induce N-cadherin upregulation (90). Notably, however, in addition to epigenetic and genetic downregulation, several other post-transcriptional events regulate junction stability and E-cadherin dynamics. A wealth of evidence points to a crucial role of E-cadherin endocytosis and recycling in tissue morphogenesis and EMT (91–96).

### E-cadherin trafficking controls the dynamics of AJs and the plasticity of epithelial tissues

E-cadherins are characterized by long extracellular and cytoplasmic domains that are primarily responsible to establish homophilic interactions between neighboring cells (97). In addition, the cytoplasmic tail associates with a variety of intracellular proteins. These latter proteins link the process of cell-cell adhesion to the actin-myosin network, to vesicle transport, and to the cell polarity machinery. A key feature of AJs is that they are dynamic. Indeed, the ability of individual AJs to be continually assembled and disassembled is key for the preservation of epithelial integrity, which must be maintained despite the constant changes in cell packing that accompany changes in tissue organization, cell division, cell death, and delamination (98–101).

**Figure 2:**
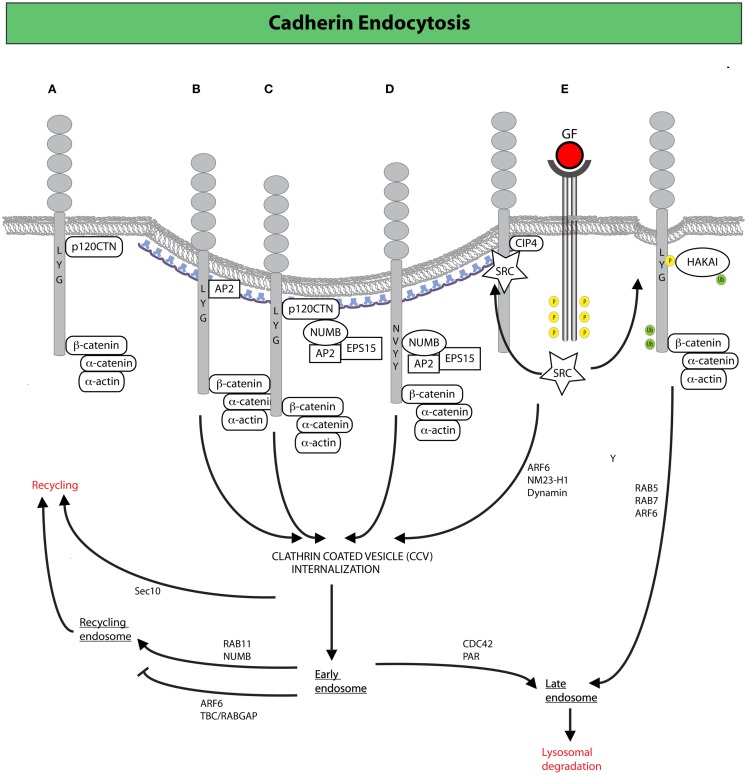
**Clathrin-mediated endocytosis (CME) and endocytic trafficking in the control of E-cadherin dynamics**. **(A)** Binding of p120CTN to the juxta-membrane region of the cytoplasmic tail of E-cadherin prevents the recruitment of endocytic adaptors favoring the stabilization of E-cadherin at the plasma membrane. Alternative recruitment of clathrin adaptors to the same region promotes CME of E-cadherin (105). **(B)** The adaptor AP-2 induces CME by displacement of p120CTN (105). **(C,D)** The endocytic protein NUMB may also drive internalization by serving as a scaffold between E-cadherin (or p120CTN) and the canonical endocytic adaptors, AP-2 and EPS15 (107, 108). NUMB may act either by **(C)** bridging together p120 and endocytic adaptors, thereby promoting the internalization of the entire E-cadherin/p120CTN complex or by **(D)** binding directly to the NVYY motif in E-cadherin, thus facilitating the internalization of p120-unbound E-cadherin, ultimately opposing p120CTN-mediated suppression of endocytosis (107, 108). **(E)** Activated SRC promotes the phosphorylation of E-cadherin enabling the binding of the ubiquitin ligase HAKAI, which induces CME, and the subsequent degradation of E-cadherin via the lysosomal route (98). Junctional activation of SRC, dependent on the endocytic F-BAR-containing protein CIP4, is also required to increase junctional tension across E-cadherin (not depicted): an event that facilitates junction dismantling and E-cadherin endocytosis (109). Following clathrin-coated vesicles (CCVs)-mediated internalization, E-cadherin can traffic through different routes, regulated by a diverse set of molecular determinants, which determine E-cadherin fate either to lysosomal-mediated degradation or late endosomal recycling back to the lateral junction, in a process that fuels AJ dynamics and the remodeling necessary for epithelial tissues homeostasis (110).

As a result of this plasticity, AJ dynamics can release stresses that have accumulated in an epithelium and accommodate morphogenetic movements. Importantly, the turnover of E-cadherin in mature epithelial tissues is primarily the consequence of active internalization and recycling processes (92, 101, 102). Studies of epithelial cells in culture revealed that impairment of CME blocks the recovery after photo-bleaching of fluorescently labeled E-cadherin (101). Similarly in the *Drosophila* pupal notum, dynamin- and actin-dependent endocytosis was shown to be required to remove surface E-cadherin and to maintain the position and stability of mature AJs (103, 104). This is consistent with the finding that a dileucine motif, which is a binding site for clathrin adaptors, is present in the cytoplasmic tail of E-cadherin and is required for internalization (105). This motif is also the binding site of p120catenin (p120CTN, not to be confused with β-catenin), an Armadillo repeat-containing junctional protein that, in polarized epithelia, prevents the access to clathrin adaptors, thereby counteracting CME (105) (Figure 2A). Not surprisingly, loss of p120CTN causes cell-cell junction disruption and is linked to EMT and invasiveness (106).

An additional mechanism through which E-cadherin membrane expression, and therefore cell-cell junction stability, can be modulated is via Presenilin (PS1)-mediated cleavage of E-cadherin. PS1, the catalytic subunit of γ-secretase, enters into an E-cadherin complex by associating with both p120CTN and β-catenin (111). Under physiological conditions, PS1 recruitment to AJ stabilizes E-cadherin junctional complexes. However, Ca^++^ influx or apoptotic stimuli can induce the junctional-restricted, proteolytic activity of the PS1/γ-secretase complex, which cleaves E-cadherin releasing an E-Cad-C-terminal fragment-β-catenin complex from the cytoskeleton that interferes with canonical WNT signaling (112). How and whether membrane and endosomal localization of PS1, which are well-established site of action of the proteolytic activity of the complex (113), participates in processing E-cadherin remains unclear, but represent another potential mechanisms though which membrane trafficking might influence E-cadherin surface level and, hence, junctional stability.

### The endocytic protein NUMB in the control of E-cadherin trafficking, EMT programs, and polarized functions

A key node in the regulation of E-cadherin trafficking is the accessibility of cytoplasmic motives (e.g., dileucine and NVYY) to p120CTN and endocytic adaptors. Among the latter, a prominent function is exerted by NUMB that, through its PTB domain, can dock directly to the NVYY motif of E-cadherin, and also bind p120CTN through its proline-rich region (107, 108) (Figures 2B,C). Through these interactions, NUMB may serve as a docking site for the recruitment of endocytic adaptors, including AP-2 and EPS15 that ultimately fine tune the rate of E-cadherin internalization (22, 114–119). Within this framework, NUMB may act on E-cadherin endocytosis in two ways: (1) bridging together p120CTN and endocytic adaptors, thereby promoting the internalization of the entire E-cadherin/p120CTN complex; (2) binding directly to the NVYY motif in E-cadherin, which is usually masked by p120CTN, and facilitating the internalization of p120CTN-unbound E-cadherin, ultimately opposing p120CTN-mediated suppression of endocytosis (107, 108). As a result of these roles, NUMB is emerging as a critical molecule in the control E-cadherin dynamics. Consistently, loss of NUMB was shown to promote EMT of MDCK cells (120), further suggesting that the simple equation reduction of E-cadherin endocytosis equals to decreased mesenchymal progression is not always valid. This may be due to the fact that silencing of NUMB was also shown to lead to (i) a lateral to apical translocation of E-cadherin and β-catenin, (ii) active F-actin polymerization, followed by mislocalization of the PAR3 and aPKC polarity proteins, (iii) a decrease in cell-cell adhesion and an increase in cell migration and proliferation: all features that mark early steps of EMT (107, 121). Furthermore, NUMB may also act at later steps of membrane trafficking controlling recycling of cargos back to the PM. Consistently, NUMB has also been implicated in the maintenance of AJs through its recycling functions, which promote the proper distribution of RAB11-endosomes containing E-cadherin in close proximity to AJs (122) (Figure 2). In summary, NUMB appears to control various critical steps (from endocytosis to recycling) in the maintenance of a proper dynamics of E-cadherin, and further to regulate the establishment of epithelial polarity. Not surprisingly, therefore, disruption of NUMB lead to loss of proper cell-cell junction, epithelial polarity, and the acquisition of mesenchymal, morphological traits. These results highlight that AJ stability, regulation of basal/apical polarity, and actin remodeling are intimately intertwined processes, whose coordinated regulation is essential for the maintenance of epithelial properties, whereas its subversion critically influences the transition to a more mesenchymal phenotype (123–127).

Mechanistically, NUMB with its pleiotropic functional roles is a paradigmatic case that provides molecular insights into how the coordination of diverse cellular polarized processes is achieved. NUMB was originally identified as a cell-fate determinant in *Drosophila* development, and underwent intense scrutiny as an inhibitor of the NOTCH receptor signaling pathway (126–138). However, NUMB does a lot more than simply regulate Notch and E-cadherin internalization, as it has been implicated in a variety of biochemical pathways connected with signaling, including NOTCH, Hedgehog, and TP53 (130–132, 139–142). At the phenotypic level, NUMB controls endocytosis (it is involved in cargo internalization and recycling), determination of polarity (it interacts with the PAR complex, and regulates adherens and tight junctions), and ubiquitination (it exploits this mechanism to regulate protein function and stability) (107, 108, 117, 124, 143–146). NUMB appears, therefore, to sit at the center of diverse cellular phenotypes, including cell-fate developmental decisions, maintenance of stem cell compartments, regulation of cell polarity and adhesion, and migration (147, 148). However, rather than exerting multiple and distinct biochemical functions, NUMB appears to interconnect these processes by integrating them at the level of endocytic network. In many respects, NUMB epitomizes the concept of endocytosis as a key infrastructure connecting diverse, necessary for the execution of polarized function. Indeed, at the cellular level, the molecular workings of Numb all seem to converge on the establishment of polarity, be this epithelial cell polarity, execution of polarized functions such as migration, or establishment of signaling directionality in asymmetric cell division. In this context, NUMB might be one of the molecular determinants integrating apparently distant polarized functions, including cell-fate determination of normal and cancer stem cells and EMT, which have indeed emerged as two faces of the same coin (15, 149).

Not surprisingly, considering its critical role in many cellular processes, subversion of NUMB has been linked to highly relevant human pathologies, including neurodegeneration and cancer (121, 148, 150–152). In this latter context, genetic evidence in *Drosophila* as well as in mouse model of lymphomagenesis indicated that NUMB is a *bona fide* tumor suppressor (132, 153–155). A similar role has also been demonstrated in human malignancy, including breast cancer, where loss of NUMB correlates with a less differentiated phenotype, expression of cancer stem cell traits and poor prognosis, salivary gland tumors, and non-small cell lung carcinomas (NSCLCs) (133, 142, 156–158).

### Inducers of EMT promote junctional remodeling via regulation of E-cadherin trafficking

Endocytic removal of E-cadherin from the cell surface is frequently and acutely induced by activation of receptor and non-receptor tyrosine kinases, as well as by stimulation with TGF-β (159–161). EMT-promoting stimuli, such as hepatocyte growth factor (HGF), EGF, or SRC activation, invariably lead to the phosphorylation of the NVYY motif of E-cadherin, impeding the interaction not only with NUMB, but also with p120CTN, while promoting the association with the ubiquitin ligase HAKAI (98) (Figure 2E). HAKAI induces E-cadherin ubiquitination and its subsequent internalization and lysosomal degradation in a process that requires the sequential activation of RAB5 and RAB7 (162) (Figure 2E). The same stimuli also activate another GTPase, ARF6, which stimulates E-cadherin endocytosis (163), in part by enhancing the activity of the nucleotide diphosphatase NM23-H1, which was recently shown to produce locally the GTP required by dynamins to execute the scission of vesicles from the PM, thus promoting internalization (164). Notably, ARF6 appears to act also at later steps of membrane trafficking, whereby its activation impedes recycling of E-cadherin (165), ensuring that cell-cell contacts will not be reformed. In this context, a key function is exerted by the RAC1 effector TBC/RABGAP, which physically links the RAC1 to ARF6 (165). Consistent with a key role exerted by ARF6 in promoting EMT, expression of an ARF6-dominant negative mutant impedes junction disassembly and prevents scattering induced by HGF and SRC, while sustained ARF6 activation disrupts the morphogenetic programs leading to the formation of glandular organization of mammary epithelial cells cultivated on 3D basement membrane (166).

The maintenance of junctional stability is also critically achieved by constant recycling of internalized AJ proteins, and of E-cadherin specifically (167–169) (Figure 2). The AJ component β-catenin was shown, for example, to interact directly with the SEC10 exocyst subunit, suggesting the possibility that β-catenin can direct the exocytosis of AJ components to specific sites on the PM (94, 170–172). Additionally, in keeping with the need for constant and polarized membrane recycling to support AJ dynamics, Rab11 (a small GTPase required for recycling from late endosomal vesicles) is also required to maintain epithelial integrity in the ventral ectoderm (173). Within this context, Rho family GTPases, and most notably CDC42, have emerged as critical molecular switches that regulate not only the formation of AJs, but also their dynamic remodeling during tissue rearrangement, by controlling multiple steps of E-cadherin trafficking and affecting the activity of polarity complexes (174, 175).

In the neuroectodermal epithelium of *Drosophila*, for example, CDC42 and PAR proteins were shown to regulate primarily the recycling of AJ components and apical polarity proteins, by promoting their progression from early to late endosome in order to maintain AJ stability in the face of cell rearrangements (176). In the developing pupal notum or dorsal thorax of the fly, CDC42 functions with PAR6/aPKC and CIP4/N-WASP (a membrane deforming and an actin nucleation promoting complex, respectively) to regulate early events in E-cadherin endocytosis, mimicking the phenotype obtained upon loss-of-function of the GTPase dynamin (103, 104). Notably, in most epithelial tissues of the fly, the apical polarity complex CDC42–PAR6–aPKC seems to induce the local activation of CIP4, the only member of the F-BAR-containing, membrane-bending family proteins expressed in *Drosophila* to drive dynamin-dependent CME of AJ material and the recycling of E-cadherin complexes (103, 104). In mammary epithelial cells, however, where three F-BAR family members exists, we recently showed that CIP4 is fully dispensable for the CDC42-mediated activation of N-WASP. CIP4 is, instead, essential for the formation of a macromolecular complex that includes E-cadherin and SRC and the localized, junctional activation of the latter protein (109) (Figure 2E). Activated SRC, in turn, may enhance actomyosin-dependent contractility across E-cadherin junctions: an event needed to dismantle AJ and associated with the subsequent removal via internalization of E-cadherin (91, 102, 160, 177, 178). Consistent with this latter scenario, removal of CIP4 impedes the increase in junctional tensile stress that is required to break junctions apart during growth factor-mediated scattering (109), adding to the emerging evidence of a tight interplay between actomyosin contractility and E-cadherin endocytosis during epithelial morphogenesis (179). Within this context, CIP4 may serve as a molecular hub interconnecting the two processes, which ultimately impact on epithelial cell cohesion, motility, and invasion (180–182). Not surprisingly, CIP4 loss has profound effect of epithelial plasticity since it increases cell compaction, delays mammary epithelial scattering and invasion into 3D matrix, and the conversion from *in situ* ductal carcinoma to invasive carcinoma in a model of breast tumorigenesis. Conversely, CIP4 up regulation in human breast cancer is associated with an aggressive phenotype and poor survival (109).

### Endocytic-dependent junctional treadmilling promotes mesenchymal motility

The acquisition of a motile mesenchymal phenotype is an important part of EMT. Also for this phenotype, endocytic circuitries are instrumental to initiate and sustain a morphological transition toward a mesenchymal migratory behavior of collective cell entities during physiological or pathological morphogenetic processes (183–187). Collective migration of cohorts, sheets, groups, or chains of cells has been commonly observed during developmental processes, in tissue renewal and in wound healing; and also it is observed also during tumor spreading [reviewed in Ref. (188, 189)]. In all these cases, and in particular in tissues of epithelial origin undergoing plastic remodeling or pathological dissemination, it is frequent to observe the emergence of cells, restricted at the migratory or invasive front and acting as leader cells, that undergo a morphological transition to mesenchymal traits. These cells can either detach from the tissues of origin and adventure into the surrounding stroma as individual mesenchymal entities or maintain cell-cell interactions, and drag, as leaders with early phenotypic mesenchymal features, an entire sheet of cells forward.

**Figure 3:**
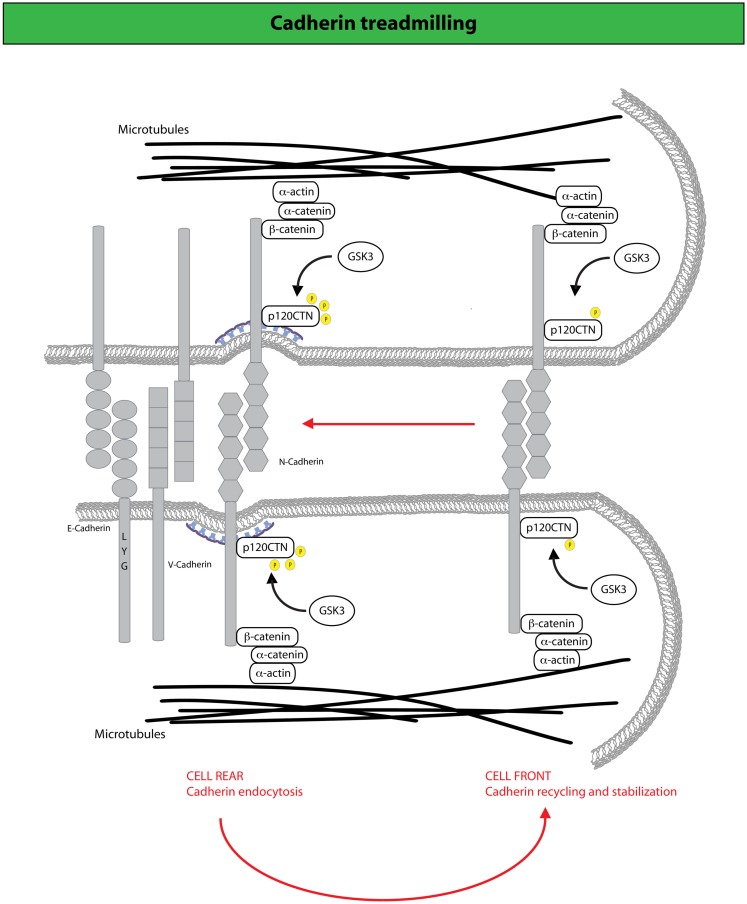
**N-cadherin treadmilling sustains collective motility**. N-cadherin dynamic sustains cell migration by constantly cycling from the cell rear, where it becomes internalized upon p120CTN phosphorylation mediated by the GSK3 kinase, and the cell front to which internalized N-cadherin is directed via endocytic recycling (190). At the cell front, N-cadherin undergoes actin-dependent retrograde flow along the lateral edges of the cell, which is driven by F-actin attachment to AJ complexes that include catenins (for example, p120-, α-, and β-catenin) and N-cadherin. Arrows indicate the direction of cadherin movement. Disruption of N-cadherin treadmilling impairs collective locomotion of astrocytes providing evidence that the retrograde movement of adherens junctions and the recycling of N-cadherin to the cell front are keys for the acquisition of collective modes of locomotion.

It has recently been shown that AJs holding together primary astrocytes undergo continuous treadmilling along the lateral sides of adjacent leading cells moving into a wound (190) (Figure 3). The treadmilling is driven by a retrograde actin flow and supported by polarized membrane trafficking of junctional N-cadherin. Indeed, N-cadherin is internalized via CME that is spatially confined to the junctions at the rear of cells, to be subsequently delivered at the leading front. Perturbation of endo/exocytic cycles arrests junctional treadmilling and impairs directional migration (190). Similar situations have been reported for E-cadherin, which undergoes apico-basal flow in epithelial cells and VE-cadherin, which is subjected to treadmilling in endothelial cells (190, 191). These findings highlight the particularly active dynamics of AJs between motile cells. AJs remodeling is, indeed, critical for the maintenance of epithelial identity, while providing a highly adaptable adhesive system when transition from a sessile to a motile state is required (97). Clearly, these transitions do not require the full genetic reprograming typically associated with complete, canonical EMT. Nevertheless, alterations in junction dynamics and composition remain a nearly invariable feature associated with the onset of EMT. Within this context, it is conceivable that harnessing endocytic networks may be a general mean to promote flexible and dynamic identity changes of epithelial tissues that may precede or accompany genetic and epigenetic reprograming.

## Concluding Remarks

Endocytic circuitries control in addition to junction stability also cell-ECM interaction, acting: (i) on the dynamic remodeling of the adhesion receptors integrins, and of focal adhesions [see Ref. (183, 192) for details]; (ii) on the acquisition (or loss) of apico-basal polarity, by regulating the appropriate spatial distribution of polarity determinants (193–195); (iii) on the regulation of polarized actin dynamics and actomyosin contractility (196, 197). Mechanistically, the impact of endocytosis on these processes, which we did not cover in this review, can be rationalized within the same basic principle that posits that the endocytic machinery defines a vast program of intracellular communication that integrates different, apparently distinct, territories of cell regulation, as according to the concept of “endocytic matrix” (19). Within this framework, it is not surprising that virtually all the key cellular processes specifying an epithelial vs. a mesenchymal identity use the endocytic matrix for their proper execution and are integrated at the level of membrane trafficking routes.

Congruently, these circuitries are frequently rewired in physiological transitional states or hijacked by malignant cells to obtain a degree of cell plasticity functional to the adaptation to micro-environmental changes. Examples of this type of “endocytic reprograming” are being documented. As discussed, TGF-β, when promoting EMT, can induce a permanent loss of cell adhesion by negatively regulating the transcription of the E-cadherin gene, but also by augmenting dynamin-dependent E-cadherin endocytosis (181, 182). In this latter case, it was shown that activin/nodal, a member of the TGF-β superfamily of cytokines, can induce the expression, among others, of two proteins regulating cell adhesion during vertebrate gastrulation (a process where EMT is necessary to complete development): (i) fibronectin leucine-rich repeat transmembrane 3 (FLRT3), a type-I transmembrane protein containing extracellular leucine-rich repeats, and (ii) Rho family GTPase 1 (RND1), an atypical member of the Rho GTPase (198), which is unable to hydrolyze GTP. These proteins interact physically and modulate cell adhesion by controlling the cell-surface levels of E-cadherin through a dynamin-dependent endocytic pathway. In keeping with this notion, a time-resolved analysis of the proteomic and phosphoproteomic changes of cultured human keratinocytes undergoing EMT and cell cycle arrest in response to stimulation with TGF-β revealed that – in addition to the expected set of cytostatic, extracellular matrix remodeling and epithelial de-differentiation gene products – a number of membrane trafficking proteins were also upregulated (1). These data reinforced the idea that biological responses to TGF-β result from extensive cross talk of different signaling pathways. They further argue that harnessing those networks, such as the endocytic matrix that integrates multiple cellular functions is instrumental to achieve plasticity of cell identity, as typified by EMT.

The identification of the various endocytic hubs at the basis of cellular plasticity is, therefore, likely to illuminate on fundamental principles on which cells and tissues are built. It will also provide potential new insights into the molecular underpinning of the development of cellular heterogeneity, which in tumor cell biology is the emerging recognized feature inextricably linked to tumor progression, dissemination, and resistance to targeted molecular therapies.

## Conflict of Interest Statement

The Review Editor Myriam Alcalay declares that, despite being affiliated to the same institution as authors Giorgio Scita, Maria Grazia Malabarba and Pier Paolo Di Fiore, the review process was handled objectively and no conflict of interest exists. The authors declare that the research was conducted in the absence of any commercial or financial relationships that could be construed as a potential conflict of interest.
